# Evaluation of Breaking Force of Different Suture Materials Used in Dentistry: An In Vitro Mechanical Comparison

**DOI:** 10.3390/ma15031082

**Published:** 2022-01-30

**Authors:** Mattia Manfredini, Susanna Ferrario, Paola Beretta, Davide Farronato, Pier Paolo Poli

**Affiliations:** 1 Maxillofacial Surgery and Odontostomatology Unit, Implant Center for Edentulism and Jawbone Atrophies, Fondazione IRCCS Cà Granda Ospedale Maggiore Policlinico, University of Milan, Via della Commenda 10, 20122 Milan, Italy; mattiamanfredinidr@gmail.com (M.M.); pierpaolo_poli@fastwebnet.it (P.P.P.); 2 Department of Medicine and Surgery, School of Dentistry, University of Insubria, 21100 Varese, Italy; davide.farronato@uninsubria.it

**Keywords:** suture materials, oral surgery, wound healing, breaking force

## Abstract

The success of surgical procedures is strictly related to the biomechanical properties of the suture. Mechanical comparisons are scarcely reported in the literature, so the purpose of the present study was to evaluate and compare the mechanical behavior of different sutures commonly used in oral surgery in terms of traction resistance. Sutures made of eight different materials were analyzed: silk (S), polyglycolide-co-caprolactone (PGCL), polypropylene (PP), rapid polyglycolide (rPGA), standard polyglycolide (PGA), polyamide (PA), polyester (PE), and polyvinylidene fluoride (PVDF). For each material, three different sizes were tested: 3-0, 4-0, and 5-0. The breaking force of each suture was assessed with a uniaxial testing machine after being immersed in artificial saliva at 37 °C. The outcomes analyzed were the breaking force, the needle–thread detachment breaking-point and the node response after forward–reverse–forward (FRF) tying when subjected to a tensile force. The 3-0 rPGA provided the maximum resistance, while the lowest value was recorded for the 5-0 PGCL. In general, 3-0 and 4-0 gauges showed non-statistically significant differences in terms of needle–thread detachment. The highest needle–thread detachment was found for the 3-0 PGA, whereas the lowest value was observed for the 5-0 PGCL. After tying the knot with an FRF configuration, the thread that showed the highest resistance to tension was the 3/0 silk, while the thread with the lowest resistance was the 5/0 silk. These data should be considered so that the operator is aware of as many aspects as possible on the behavior of various materials to ensure successful healing.

## 1. Introduction

Generally, proper closure and stabilization of wound margins in their desired position are mandatory to obtain successful surgical procedures. In oral surgery, wound healing is largely dependent upon the formation, organization, and stability of the blood clot during the early post-operative phases. This allows the formation of a matrix that connects the edges of the wound, enhances cells adhesion, supports neoangiogenesis, and restores the resistance of the tissues to functional stress [1]. Impaired clot adhesion may weaken the tensile strength of the wound during the early healing events leading to an increased susceptibility to soft tissue tearing and dehiscence [2]. Since the dehiscence of the surgical wound is frequently related to suture tension, it is evident that biomechanical properties of the sutures may play a critical role to counteract the physiological tensile forces arising on wound margins. In these terms, suture characteristics can be decisive factors for preventing dehiscences of surgical wounds. Amongst them, the tensile strength (TS) of suture materials is an important mechanical property, defined as the ratio of maximum load a suture can withstand without breaking while being stretched to the original cross-sectional area of the given material [3]. An adequate TS of a suture material is of utmost importance for the proper coaptation of the flaps until healing has completed. Moreover, TS needs to be maintained particularly within the first 2 post-operative weeks, because suture materials tend to lose between 70% and 80% of their initial strength [4]. Therefore, the necessary initial TS in a horizontal plane must be guaranteed to avoid breaking the suture material [5]. Conversely, sutures with insufficient TS during the healing phase could break, causing poor adaptation of the wound margins and hematoma, which separates the healing flaps from the underlying bone. This may result in observable tissue scarring and subsequent deterioration of the affected site [6]. The TS of a suture material is even more important in oral surgery, where, differently from other parts of the body, the constant presence of saliva, and functions related to tongue mobility, speech, mastication, and swallowing, may negatively affect the flap tension. In this matter, a relationship between wound-closing tension and TS was demonstrated. Increasing closing tension may lead to higher TS and consequently to a stronger scar with increased collagen deposition [7]. Sutures can be categorized according to several criteria based on the materials used for the production of the suture surgical threads, on the base of their origin and their biological behavior, and finally on the base of their structure. Each of them has its own TS that should be compared among the different suture materials in order to select the appropriate type of suture according to the expected tensile force exerted on the flaps. However, to the best of our knowledge, only a few studies have compared the TS of different suture materials under simulated oral conditions for clinical applications in dentistry.

Moreover, in everyday clinical practice, sutures are selected from the clinician depending on the type of intervention they are required for; amongst all the features, TS and some few factors play a more important role than others, such as plaque retainment capacity, width, length, and dimension of the gauge, and reabsorption activity. Even if most surgical protocols require specific sutures by their original description, generally suture choice mainly depends on the surgeon’s preferences.

In view of the above, the aim of the present study was to evaluate and compare in vitro the breaking force (BF), as an indirect expression of the TS, the needle–thread detachment breaking point, and the behavior of the node after forward–reverse–forward (FRF) tying when subjected to a tensile force of different suture materials commonly used in the daily practice during oral surgery procedures, in order to support the clinician’s choices by providing more detailed information on these specific features.

## 2. Materials and Method

The study consisted of eight different types of suture materials (LORCA MARÍN S.A., Murcia, Spain): silk (S), multifilament polyethylene (PE), monofilament polyvinylidene fluoride (PVDF), pseudo-monofilament polyamide (PA), monofilament polypropylene (PP), rapid synthetic multifilament polyglycolic acid (rPGA), synthetic multifilament polyglycolic acid (PGA), and pseudo-monofilament polyglycolic acid (PGCL). Three gauges of each type were used (3-0, 4-0, and 5-0) providing a total of 24 different types of threads. The materials and gauges were selected based on their common usage in oral surgery. Each sample was 20 mm long. The sutures were tested using a dual-column bench-mounted material testing machine (LR30K Plus, LLOYD Instruments, Bognor Regis, UK) with a minimum load resolution of 10^−4^ N. BF was analyzed and defined as follows: the level of stress at which the tested material failed.

A commercial artificial saliva (BioVision, Inc., Milpitas, CA, USA) was employed. It was maintained in an incubator at a temperature of 37 °C, and the composition was as follows: potassium chloride (1.20 g), sodium chloride (0.85 g), sodium phosphate dibasic (2.50 g), calcium chloride (0.15 g), magnesium chloride (0.15 g), carboxymethylcellulose (3.10 g), sorbitol (0.75 g), methyl-parahydroxybenzoate (0.25 g), and purified water (1000 mL).

Due to the effect of many variables on the salivary composition, it is impossible to create a single model of artificial saliva that is universally applicable [8]. The authors selected this specific substitute because it appeared to provide the best properties for the applicational requirements of the study.

**Figure 1:**
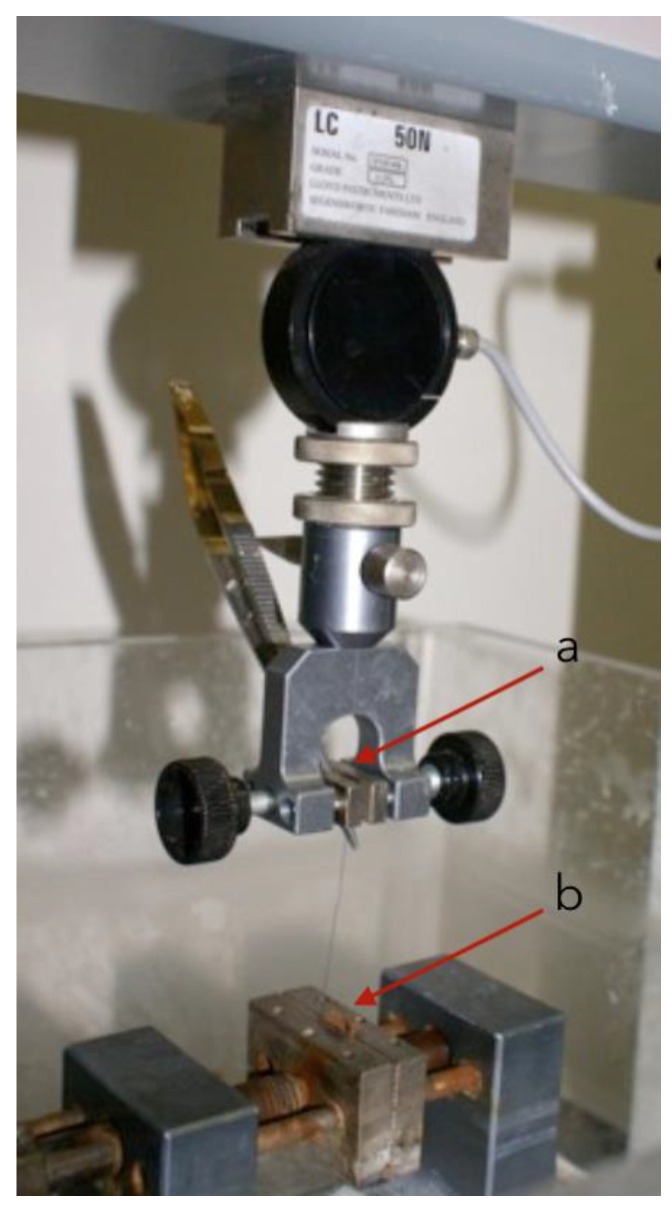
Experiment procedure: thread is attached to a Castroviejo, which is attached to the mobile clamp of the machine (**a**); the lower head of the thread is held in place by a narrow copper plate in the static clamp of the machine (**b**).

Each sample was kept in a container approximately filled with artificial saliva. One end was secured to a straight Castroviejo-type needle holder (Hu-Friedy Mfg. Co., LLC, Chicago, IL, USA) with tungsten clamps fitted to the mobile clamp of the testing machine. The other end was secured to a narrow copper plate attached to the static metal clamp. The procedure is illustrated in Figure 1.

As copper is a ductile and smooth material, the pressure was evenly distributed over the entire filament structure without creating any injury near the serrated clamp. This method allows avoiding the use of a knot, which would act as a stress concentration. Even if a knot is always present when suturing in the oral cavity, the authors preferred to conduct the test without knotting, in order to obtain pure analysis of failure force of the suture. The knot test (failure load, knot slippage, and knot breakage) was conducted separately using only a single knot type, with an FRF configuration, which is commonly used in clinical practice. Further studies will be required to better understand the role and the resistance of any suture’s knots when put under tension. The fixed distance between the needle holder and the metal clamp was 22 mm. Needle–thread detachment test was conducted in a similar way to the previous one: the needle was kept at the copper plate, from the same system of static metal clamp, and with the curved surface projecting downward. The wire was then clamped at its end with the same modality as described before and orientated perpendicularly from the edge of the needle. All the tests were conducted at room temperature, at a traction speed of 10 mm/min, and with a maximum tensile load of 10 kg. Knot resistance test was also conducted similarly: a 10-cm-long section was cut for each strand and the knot was tied with an FRF configuration around a 20-mm-diameter cylinder using the Castrovejo needle holder. The lower end of the wire was attached to the copper plate in the static clamp and the upper end to the Castrovejo, as described above, trying to keep the knot in the center. Each wire was dipped in artificial saliva as described above. Five tests were carried out on each knot and on each strand, for a total of 120 tests. The dynamometer pulled each knot in the manner described above until the knot was completely closed or the thread was frayed/broken. Each specimen was stretched to failure under the uniaxial load, in order to evaluate the BF, the needle–thread detachment breaking point, and the knot resistance. The maximum load was recorded in Newtons (N) and tabulated for analysis. In case of suture slippage, the result was nullified, and the test was repeated with a new sample.

A preliminary sample size calculation was performed with dedicated software (G*Power 3.1, Heinrich-Heine University, Dusseldorf, Germany) [9]. The results of the tensile load reported by [10] were taken as a reference according to [11]: 14.3 ± 1.6 N for nylon, and 10.0 ± 1.2 N for silk. The sample size required to achieve a power of 1-β = 0.80 for the one-sided chi-square test at level α = 0.05 with an effect size of 3.04 was n = 3 tests per group. The statistical analysis was performed with the IBM SPSS Statistics 24.0 software (IBM Corp., Armonk, NY, USA). A preliminary Shapiro–Wilk test was used to assess the normality of the data distribution of suture materials and gauges. All the differences were normally distributed (*p* > 0.05), therefore parametric tests were used. Afterwards, the data were initially submitted to the one-way analysis of variance (ANOVA) to determine if the materials and the gauges were different with respect to the BF and the needle–thread detachment breaking point. In case of significant differences, multiple comparisons among groups were performed using the Tukey’s post hoc test. The level of significance was set at α = 0.05. Values were expressed as mean ± standard deviation.

## 3. Results

### 3.1. Breaking Force

**Figure 2:**
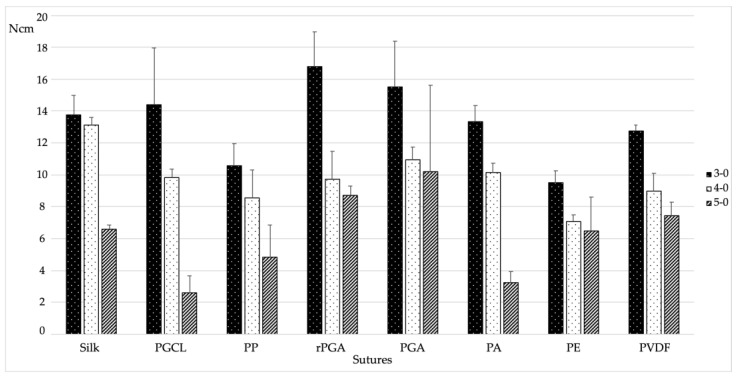
Bar chart of the Breaking Force results (N).

Overall, each type of thread underwent five consecutive tests to evaluate the BF. The results are summarized in Table 1 and illustrated in Figure 2.

When analyzing the descriptive statistics, the 3-0 gauge showed the highest BF values compared to the other gauges. The highest BF was found for the 3-0 rPGA, with a value of 16.81 ± 2.18 N (Figure 2). Considering equal gauges, this difference was statistically significantly higher compared to the PP (*p* < 0.001), PE (*p* < 0.001), and PVDF (*p* = 0.18) materials. Conversely, S, PGCL, PGA, and PA showed non-statistically significant differences when compared to rPGA. On the other hand, the lowest BF value was observed for the 5-0 PGCL, with a value of 2.62 ± 1.06 N (Figure 2). In this respect, considering equal gauges, the only similarity was found with the PA (*p* = 0.942). The rest of the suture materials showed statistically significantly higher values compared to the above-mentioned threads. For all the suture materials except for the PGA, the 3-0 gauge showed a statistically significantly higher BF compared to the 5-0 gauge. In the PGA group, although higher values were observed for the 3-0 gauge compared to the 5-0 gauge, no statistically significant differences were found. A statistically significant decreasing trend was observed for the PA and PVDF groups, in which the BF values decreased significantly as the gauge reduced.

### 3.2. Needle–Thread Detachment

**Figure 3:**
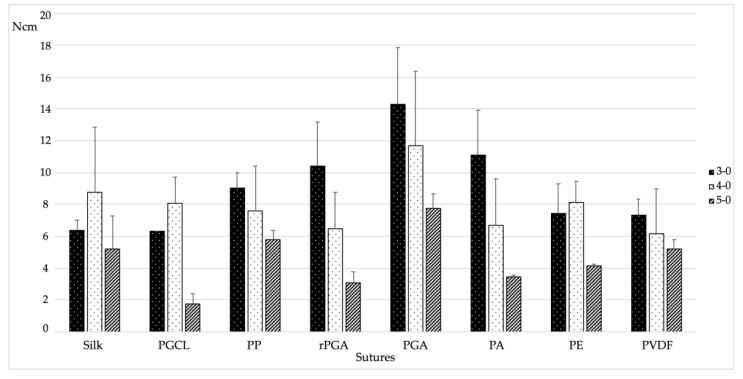
Bar chart of the needle–thread breaking point tests results (N).

Needle–thread detachment breaking point was considered as the force at which the needle detaches from the suture’s wire. Overall, each type of thread underwent three consecutive tests to evaluate the needle–thread detachment breaking point. The results are summarized in Table 2 and illustrated in Figure 3.

When analyzing the descriptive statistics, the results varied among different gauges. Indeed, quite surprisingly, for the S, PGCL, and PE, the 4-0 gauge showed higher needle–thread detachment breaking point compared to the 3-0 gauge of the same material. It must be noted, however, that this difference was never statistically significant. More in general, for all the tested materials, 3-0 and 4-0 gauges showed non-statistically significant differences in terms of needle–thread detachment breaking point. Conversely, in all the tested materials, the 5-0 gauge showed the lowest resistance to needle–thread detachment. The highest needle–thread detachment breaking point was found for the 3-0 PGA, with a value of 14.29 ± 3.57 N (Figure 3). Considering equal gauges, PP, rPGA, and PA performed similarly, while S, PGCL, PE, and PVDF showed statistically significantly lower values (*p* = 0.005, 0.013, 0.008, and 0.14, respectively). On the other hand, the lowest needle–thread detachment breaking point was observed for the 5-0 PGCL, with a value of 1.78 ± 0.62 N (Figure 3). In this respect, considering equal gauges, rPGA, PA, and PE showed similar results, whereas statistically significant higher values were found for S (*p* = 0.006), PP (*p* = 0.002), PGA (*p* < 0.001), and PVDF (*p* = 0.006).

### 3.3. Knot Test

The failure load of the knot was calculated as the maximum tension the material can withstand without tearing. In addition, knot slippage was defined as a knot that slips easily along the cord or line around which it is made, and knot breakage as the point at which the material breaks under loading.

Due to the large number of results, mainly intergroup values were analyzed. In fact, for each type of wire in each diameter, five tests were carried out for a total of 120 experiments.

**Figure 4:**
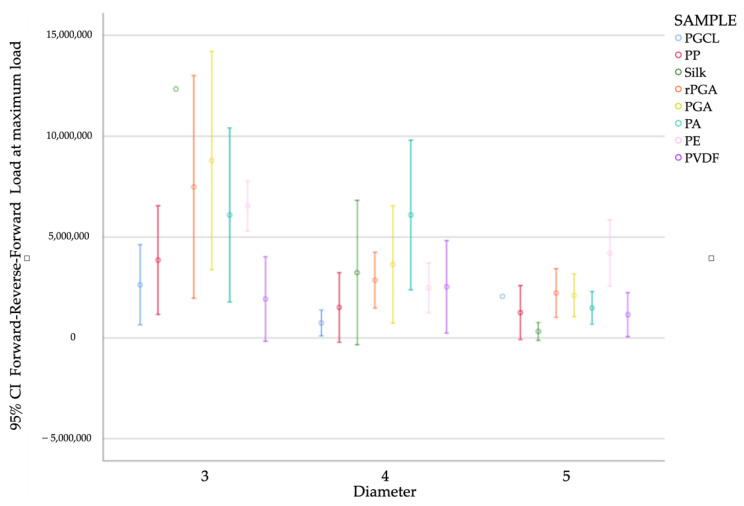
Bar chart of the knot resistance tests results (N).

In general, the results showed that as the diameter decreases, irrespective of the wire, the force required also decreases. The results are summarized in Figure 4.

In diameter 3/0, the thread that showed the highest failure load was silk (12 ± 0.58 N) compared to PVDF (1.92 ± 2.27 N), which was the thread that showed the least failure load. The result was statistically significant (*p* = 0.004).

Silk was also the thread that showed more variability among the three diameters analyzed with statistically significant results (*p* = 0.006). In diameter 3/0, as described above, the average strength was 12 ± 0.58 N, in diameter 4/0 the average strength was 3.23 ± 3.41 N, and in diameter 5/0 the average strength was 0.31 ± 0.36 N.

The wires that demonstrated low force values even in their largest diameter (3/0) were PGCL and PVDF, with 2.63 N and 1.92 N, respectively.

In diameter 4/0, the thread that showed the highest failure load was PA (6 ± 3 N) compared to PGCL (0.74 ± 0.61 N), which was the thread that showed the least failure load. The result was statistically significant (P = 0.011). Another material that showed good tensile strength was PGA (6.63 ± 2.77 N). However, the result was not statistically significant.

In diameter 5/0, the thread that showed the highest failure load was PE (4.2 ± 1.32 N) compared to silk (0.31 ± 0.16 N), which was the thread that showed the least failure load. Another material that showed good tensile strength was PGA (6.63 ± 2.77 N).

## 4. Discussion

The present study aimed to test in vitro the BF of different suture materials frequently used in oral surgery due to their versatility and popularity. Suturing, intended as the surgical act performed to approximate the flaps, is mandatory to stabilize the wound margins effectively until the healing process confers the intrinsic force needed to maintain flap coaptation, without the necessity of a mechanical support. Furthermore, the surgical suture aims to promote the cicatrisation process, control the hemostasis, stabilize the soft tissues in the desired position, and protect the wound by external contaminants, which in turn improves the post-operative patient morbidity [12]. Therefore, it can be deduced that the selection of the correct suture material is of utmost importance to achieve an ideal healing of the soft tissues. This is even more important in the oral cavity, where the presence of acidic and organic fluids, an extremely complex oral microbiome, together with inevitable functional movements and impaction of food debris, may severely compromise the first-intention healing of the surgical wound. In these terms, the BF of a suture material plays a pivotal role in the ability to withstand stress during knotting and in protecting the wound during an extended period of healing [13]. On the other hand, the needle–thread detachment and knot resistance analysis can provide important information to the clinician; a higher breaking point results in higher resistance, which reduces the risk of complications during the act of suturing. In fact, the loss of a gauge in the oral cavity represents a complication that may endanger the patient, while resulting in a more difficult suturing phase for the operator. In the present study, rPGA, silk, and PGA showed the highest BF in the 3-0, 4-0, and 5-0 groups, respectively. Thus, such materials can be recommended in those clinical situations that require high BF. This complies favorably with Minozzi and co-workers who stated that in anatomic regions such as the oral mucosa that demand higher TS, the multifilament suture material is preferred [12]. Considering the measured values, in all the suture materials tested, except PGA, it emerged that as the gauge decreases, the BF detected decreases correspondingly. This is in accordance with the study carried out by Kim and co-workers [10] who showed that resorbable sutures, after being immersed in artificial saliva at 37 °C, were able to maintain their tensile capacity. Indeed, the highest resistance values recorded corresponded to resorbable materials. All 3-0 filaments that were considered, including both resorbable and non-absorbable, showed more or less the same TS with values ranging from 10 to 20 N.

The results observed herein showed that the PGA was the material with the highest mean BF in the three gauges considered. This is in accordance with several studies [14,15] that defined the PGA as a manageable material, with an excellent initial TS and a lower tissue reaction. Moreover, in the majority of suture materials analyzed in this study, statistically significant differences between 4-0 and 5-0 gauges of the same material were found. These data are in line with the study carried out by Khiste and colleagues [16] who observed a higher TS in the 4-0 caliber compared to the 5-0 caliber, even after a 14-day period. The present study has an intrinsic methodological limitation due to the fact that the experimentation was performed in vitro and not in vivo. In the study carried out by Ferguson and coworkers [17], the re-absorbable sutures immersed in saliva inside the oral cavity for a certain period tended to lose their TS. This is due to the mechanism of biodegradation that is accelerated by the saliva itself.

Finally, considering the measured values, in all the suture materials tested, except PGCL, it emerged that as the gauge decreases, the knot resistance detected decreases correspondingly. Our results are in line with those obtained in another study [18], which showed that silk is the strongest material in the knot configuration analyzed herein (FRF).

Therefore, further studies are necessary to better understand the behavior of different suture materials during their clinical use. It must be clarified that the main goal of the present study was not to classify the best suture, but to provide the clinician with useful information related to the different types of suture materials. These data can be taken into consideration so that the surgeon is aware of as many aspects as possible concerning the behavior of the available materials, in order to choose the ideal thread according to the type of surgery that has been performed.

## 5. Conclusions

Within the limitations of the present in vitro study, the results showed different behaviors among the materials and the calibers evaluated. The 3-0 gauge showed the highest BF values compared to the other gauges. The highest BF was found for the 3-0 rPGA. In general, rPGA, silk, and PGA showed the highest BF in the 3-0, 4-0, and 5-0 groups, respectively. Thus, such materials can be recommended in those clinical situations that require high TS.

## Figures and Tables

**Table 1:** Breaking Force values (N) observed for each type of thread expressed as mean ± standard deviation.

Type of Suture	Gauge			*p*-Value		
Material	3-0	4-0	5-0	3-0 vs. 4-0	3-0 vs. 5-0	4-0 vs. 5-0
S	13.75 ± 1.21	13.13 ± 0.49	6.57 ± 0.29	0.44	<0.001 *	<0.001 *
PGCL	14.4 ± 3.57	9.83 ± 0.51	2.62 ± 1.06	0.08	<0.001 *	0.006 *
PP	10.6 ± 1.37	8.56 ± 1.75	4.83 ± 2.03	0.18	0.002 *	0.03 *
rPGA	16.81 ± 2.18	9.72 ± 1.78	8.69 ± 0.63	<0.001 *	<0.001 *	0.6
PGA	15.52 ± 2.84	10.93 ± 0.83	10.19 ± 5.45	0.14	0.08	0.94
PA	13.32 ± 1.02	10.17 ± 0.56	3.25 ± 0.68	<0.001 *	<0.001 *	<0.001 *
PE	9.5 ± 0.78	7.07 ± 0.43	6.47 ± 2.12	0.07	0.18 *	0.784
PVDF	12.74 ± 0.39	9 ± 1.07	7.45 ± 0.84	<0.001 *	<0.001 *	0.02 *

* = Statistically significant difference.

**Table 2:** Needle–thread detachment breaking point values (N) observed for each type of thread expressed as mean ± standard deviation.

Type of Suture	Gauge			*p*-Value		
Material	3-0	4-0	5-0	3-0 vs. 4-0	3-0 vs. 5-0	4-0 vs. 5-0
S	6.4 ± 0.62	8.79 ± 4.08	5.22 ± 2.07	0.22	0.31	0.12
PGCL	6.35 ± 0.00	8.1 ± 1.6	1.78 ± 0.62	0.24	0.01 *	0.004 *
PP	9.05 ± 0.94	7.6 ± 2.84	5.77 ± 0.62	0.6	0.13	0.46
rPGA	10.42 ± 2.73	6.48 ± 2.29	3.06 ± 0.71	0.13	0.01 *	0.19
PGA	14.29 ± 3.57	11.71 ± 4.64	7.74 ± 0.93	0.61	0.09	0.38
PA	11.11 ± 2.81	6.7 ± 2.9	3.45 ± 0.13	0.13	0.01 *	0.27
PE	7.42 ± 1.88	8.14 ± 1.34	4.15 ± 0.12	0.79	0.04 *	0.02 *
PVDF	7.34 ± 0.99	6.15 ± 2.81	5.22 ± 0.6	0.7	0.36	0.79

* = Statistically significant difference.

## Data Availability

No new data were created or analyzed in this study. Data sharing is not applicable to this article. The corresponding author remains available for any further clarification.
